# Kaempferol inhibited VEGF and PGF expression and *in vitro* angiogenesis of HRECs under diabetic-like environment

**DOI:** 10.1590/1414-431X20165396

**Published:** 2017-03-02

**Authors:** X.H. Xu, C. Zhao, Q. Peng, P. Xie, Q.H. Liu

**Affiliations:** 1 Department of Ophthalmology, The First Affiliated Hospital of Nanjing Medical University, Nanjing City, Jiangsu Province, China; 2 People's Liberation Army 454 Hospital, Nanjing City, Jiangsu Province, China

**Keywords:** Diabetic retinopathy, Kaempferol, VEGF, PGF, Anti-angiogenic

## Abstract

Diabetic retinopathy (DR) is one of the common and specific microvascular complications of diabetes. This study aimed to investigate the anti-angiogenic effect of kaempferol and explore its underlying molecular mechanisms. The mRNA expression level of vascular endothelial growth factor (VEGF) and placenta growth factor (PGF) and the concentrations of secreted VEGF and PGF were measured by qTR-PCR and ELISA assay, respectively. Human retinal endothelial cells (HRECs) proliferation, migration, and sprouting were measured by CCK-8 and transwell, scratching wound, and tube formation assays, respectively. Protein levels were determined by western blot. High glucose (25 mM) increased the mRNA expression levels of VEGF and PGF as well as the concentrations of secreted VEGF and PGF in HRECs, which can be antagonized by kaempferol (25 µM). Kaempferol (5-25 µM) significantly suppressed cell proliferation, migration, migration distance and sprouting of HRECs under high glucose condition. The anti-angiogenic effect of kaempferol was mediated via downregulating the expression of PI3K and inhibiting the activation of Erk1/2, Src, and Akt1. This study indicates that kaempferol suppressed angiogenesis of HRECs via targeting VEGF and PGF to inhibit the activation of Src-Akt1-Erk1/2 signaling pathway. The results suggest that kaempferol may be a potential drug for better management of DR.

## Introduction

Diabetic retinopathy (DR) is one of the common and specific microvascular complications of diabetes and is also the leading cause of new-onset blindness in the developed world (1,2). Retinal neovascularization is considered an important factor in the pathogenesis of DR (3). Vascular endothelial growth factor (VEGF) is a signal protein produced by cells that stimulate vasculogenesis and angiogenesis. The levels of VEGF were found to be increased in the aqueous humour of DR patients and in endothelial cells exposed to high glucose (4–6). In the rat diabetic animal model, increased levels of VEGF and upregulation of VEGF receptor-1 (VEGFR-1) and VEGF receptor-2 (VEGFR-2) were also detected in the retina (7). Activation of these receptors by VEGF promotes angiogenesis by inducing migration and sprouting of endothelial cells. Therefore, VEGF has been suggested to be an important mediator in the progression of DR, and anti-VEGF agents have been used as a strategy for the management of DR.

Placental growth factor (PGF) is a protein that is encoded by the *PGF* gene, and it is a member of the VEGF superfamily (8). Like VEGF, PGF has also been found to play an important role in angiogenesis, and especially in embryogenesis (9). PGF not only activates its own signaling via VEGFR-1 that is independent of VEGFA, but also enhances VEGF signaling by displacing VEGFA from VEGFR-1 to VEGFR-2, which subsequently amplifies VEGFR-2 signaling (10). Increased levels of PGF have also been found in the vitreous of DR patients (11), which may indicate that PGF is involved in the progression of DR. Therefore, targeting multiple VEGF family members may provide therapeutic strategies for the treatment of DR.

Kaempferol (3,4′,5,7-tetrahydoxyflavone), one the most commonly found dietary flavonoids, has been isolated from grapefruit, tea, broccoli, and other plant sources (12). The anti-cancer effects of kaempferol have been demonstrated in various studies. Its anti-cancer functions have been proposed to be via inhibiting DNA synthesis including nuclear DNA degradation, and inhibiting kinase activities (13,14). A recent study also suggested an anti-angiogenic role of kaempferol by inhibiting VEGF in ovarian cancer via the ERK-NFκB-cMyc-p21 pathway (15). In a transgenic-zebrafish model, kaempferol suppressed angiogenesis through inhibiting VEGFR2 expression, which can be enhanced by FGF inhibition (16). In addition, studies also indicate that oxidative stress may play a central role in the pathogenesis of diabetic retinopathy (17), and several types of natural products with anti-oxidative property have been shown to be beneficial to diabetic retinopathy. For example, anthocyanins with potential properties in protecting against oxidative stress in some degenerative diseases also showed potential beneficial effects on diabetic retinopathy (18). Another study also demonstrated that adults with diabetes consuming more flavonoid-rich fruits and vegetables have reduced odds of diabetic retinopathy. A further study suggests that polyphenols with anti-oxidative properties may target Nrf2 as a therapeutic strategy for diabetic retinopathy (19). Kaempferol, as a natural flavonol, may be also beneficial to diabetic retinopathy due to its anti-oxidative property (20). Also, kaempferol exerts an anti-inflammatory function by modulation of gene expression as well as pro-inflammatory enzyme activities (12,21). In addition, studies have demonstrated the cardio-protective, anti-bacterial and anti-viral effects of kaempferol (12). However, whether kaempferol has an anti-angiogenic effect on endothelial cells is unknown.

In the present study, we investigated the anti-angiogenic effect of kaempferol on HRECs under high glucose condition.

## Material and Methods

### Cell culture

HRECs were purchased from ATCC, cultured in a human microvascular endothelial medium (Cell Applications, Inc., USA) and maintained at 37°C in a humidified 5% CO_2_ incubator. To maintain uniform conditions, all experiments were carried out using passage 3-6 HRECs.

### Treatment of HRECs

For the time-dependent study, HRECs were treated with 25 mM glucose for 12, 24, or 48 h; HRECs that received 5 mM normal glucose were used as negative control. For the kaempferol treatment study, HRECs were divided into 5 mM normal glucose, 25 mM glucose and 30 mM glucose plus different concentrations of kaempferol (5–25 µM; Sigma, USA). The HRECs were split at 90% confluence and sub-cultured in 96-well plates or 6-well plates according to the appropriate assay conditions. After cultured in the above media for 12, 24, or 48 h, HRECs were used for the subsequent experiments.

### RNA preparation and qRT-PCR analysis

Total RNAs were isolated from cells using Trizol reagent (Invitrogen, USA). The TaqMan Reverse Transcription Kit (Takara, China) was used to obtain cDNA for mRNA detection. For VEGF and PGF mRNA, qRT-PCR was performed using SYBR Green PCR Kit (Takara) according to manufacturer instructions. GAPDH was used as internal control. The primers for VEGF mRNA were: forward, 5′-TGCCATCCAATCGAGACCCTG-3′ and reverse, 5′-GGTGATGTTGGACTCCTCAGTG-3′; the primers for PGF mRNA were: forward, 5′-AAGATGCCGGTCATGAGGC-3′ and reverse, 5′-CTGCATGGTGACATTGGC-3′. Data are reported as fold changes relative to GAPDH calculated based on the following formula: RQ=2^-ΔΔCt^.

### ELISA for VEGF and PGF

The concentrations of secreted VEGF and PGF in the media were measured by the human VEGF165 and PGF enzyme-linked immunosorbent assay (ELISA) kits (R&D Systems, USA) according to manufacturer instructions (22).

### Cell proliferation assay

HRECs were seeded onto each well of a 96-well plate, allowed to adhere for 24 h, and were cultured in serum-free media for starvation for 24 h. The cells were then treated with different concentrations of glucose (5 and 30 mM) with or without kaempferol (5–25 µM) for 24 h, and the proliferative activity was determined by CCK-8 assay according to manufacturer instructions. In brief, 10 µL CCK-8 was added to each well followed by incubation for an additional 2–4 h, and the absorbance at a 450 nm wavelength was detected.

### Transwell assay

The bottom of 24-well transwell inserts (8 μm pore size, Costar, USA) was coated with 2 μg/mL of fibronectin at 4°C overnight. The following day, 10^5^ HRECs/mL were seeded on the upper chamber of inserts with serum-free medium. After being cultured with different media for 24 h, HRECs on the upper side of the insert were scraped off gently with a cotton swab, and HRECs on the bottom side of the insert were washed with PBS, fixed with 4% paraformaldehyde and then stained with hematoxylin and eosin. The number of cells that migrated to the bottom side of the insert was counted under a microscope.

### Scratching wound assay

HRECs were starved with serum-free medium for 12 h when the HRECs had grown to 90% confluence in the 6-well plates. When the HRECs had grown to over-confluence, a wound of appropriate width was made with a 200 µL tip. Then, the HRECs were washed with sterile 1×PBS three times to remove the floating cells, incubated with different media for 24 h, and then cultured in the 6-well plate at 37°C in a 5% CO_2_ incubator. The migration monolayer was photographed at 0 and 24 h, and the migration distance was measured.

### Tube formation assay

For the tube formation assay, the 2:1 mixture of chilled Matrigel (Beckton Dickinson Biosciences, UK) and endothelial growth medium were dispensed into pre-chilled wells of a 12-well plate, and then incubated at 37°C in a 5% CO_2_ incubator for 30 min to solidify the Matrigel. HRECs that had been cultured under appropriate media for 48 h were seeded on the solidified Matrigel at a density of 10^6^ cells/well. The plates were then placed in a humidified atmosphere of 5% CO_2_ and 95% air at 37°C for 8 h to allow the formation of a capillary-like structure. The plates were photographed, and the number of capillaries formed was qualitatively assessed using Image-Pro Plus 6.0 software (Media Cybernetics, USA).

### Western blot

HRECs were lysed in lysis buffer containing protease inhibitor, and the protein concentration was measured using a Bio-Rad Protein Assay Kit (Bio-Rad Laboratories, USA) according to manufacturer instructions. Proteins were then separated by sodium dodecyl sulfate-polyacrylamide gel electrophoresis, then transferred to a polyvinylidene fluoride membrane. The following primary antibodies were used: rabbit anti-PI3K (1:1000; Abcam, USA), rabbit anti-Erk1/2 (1:1000; Abcam), rabbit anti-phosphorylated Erk1/2 (p-Erk1/2, 1:1500; Santa Cruz Biotechnology, USA), rabbit anti-Src (1:500; Abcam), rabbit anti-phosphorylated Src (p-Scr, 1:1000; Abcam), rabbit anti-Akt1 (1:2000; Abcam), rabbit anti-phosphorylated Akt1 (p-Akt1, 1:1500; Abcam), and mouse anti-β-actin (1:6000; Santa Cruz Biotechnology). Membranes were then incubated with the horseradish peroxidase-conjugated secondary antibodies (1:4000; Abcam). The membranes were exposed using a ChemoDoc XRS detection system (Bio-Rad, Italy).

### Statistical analysis

Statistical analyses were performed using SPSS 15.0 software (SPSS Inc., USA). Data are reported as means±SD, and the differences among treatment groups were compared by one-way ANOVA, followed by Dunnett's multiple comparison test, as appropriate. Differences were considered to be significant when P<0.05.

## Results

### Effect of high glucose on VEGF and PGF expression

**Figure 1:**
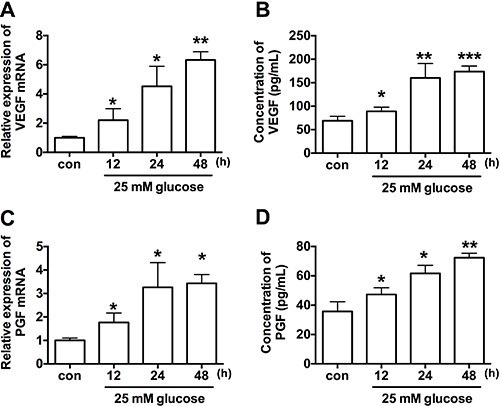
Effect of high glucose (25 mM) for 12, 24, or 48 h on mRNA expression levels of (*A*) vascular endothelial growth factor (VEGF) and (*C*) placenta growth factor (PGF) expression in human retinal endothelial cells (HRECs measured by qRT-PCR. Protein concentrations of VEGF (*B*) and PGF (*D*) in the media were measured by ELISA. Data are reported as means±SD (n=3). *P<0.05, **P<0.01, ***P<0.001 compared to control (con) groups (one-way ANOVA, followed by Dunnett's multiple comparison test).

The results of qRT-PCR showed that high glucose treatment significantly increased the mRNA expression levels of VEGF and PGF in a time-dependent manner when compared to control group (Figure 1A and B). The concentrations of secreted VEGF and PGF were measured by ELISA kit, and high glucose treatment also increased protein concentrations of VEGF and PGF in the media in a time-dependent manner when compared to control group (Figure 1C and D).

### Effect of kaempferol on cell proliferation, migration and tube formation of HRECs under high glucose condition

**Figure 2:**
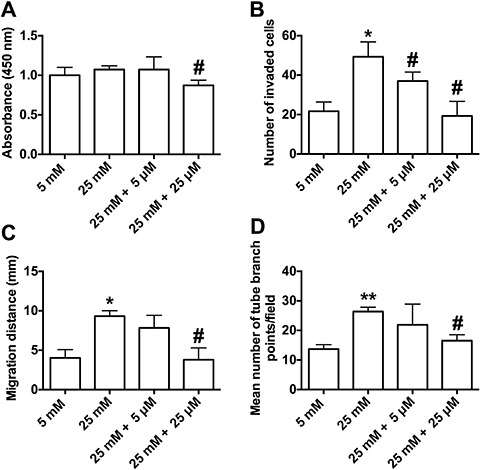
Effect of kaempferol in human retinal endothelial cells (HRECs) angiogenesis induced by high glucose (25 mM) alone or high glucose plus 2 concentrations of kaempferol (5 and 25 µM) for 24 h. *A*, HREC proliferation ability measured by CCK-8 assay. *B*, HREC invasion ability measured by cell invasion assay. *C*, HREC migration ability measured by wound scratching assay. *D*, HREC tube formation determined by Matrigel assay. Data are reported as means±SD (n=3). *P<0.05, **P<0.01 compared to 5 mM; ^#^P<0.05 compared to 25 mM group (one-way ANOVA, followed by Dunnett's multiple comparison test).

The *in vitro* CCK-8 assay showed that high glucose treatment had no significant effect on the proliferation ability of HRECs compared to 5 mM glucose treatment group; the proliferative ability of HRECs was inhibited by treatment with high glucose 25 µM plus kaempferol (Figure 2A).

To further examine if kaempferol affected the angiogenesis process of HRECs under high glucose condition, we performed transwell migration and wound scratching assays to assess the effect of keampferol on HREC migration. For the transwell migration assay, the number of cells that migrated through the transwell under 25 mM high glucose condition was more than those under 5 mM normal glucose condition, and this migration was significantly suppressed by the treatment with 25 µM kaempferol for 24 h (Figure 2B). Similar results were observed in the wound scratching assay: HRECs under 25 mM high glucose condition showed an enhanced ability to migrate with accelerated wound closure, while the wound area remained wide in HRECs under 5 mM normal glucose, and high glucose plus 25 µM kaempferol conditions (Figure 2C). Our results indicated that kaempferol inhibited glucose-induced migration of HRECs.

To examine the effect of high glucose and kaempferol on angiogenesis, we also performed Matrigel assay to measure the tube formation of HRECs. Compared to 5 mM normal glucose, high glucose significantly increased the number of capillary-like structures, while co-treatment with 25 mM high glucose and 25 µM kaempferol produced a lower number of capillary-like structures compared to 25 mM high glucose treatment group (Figure 2D). The results suggested that kaempferol inhibited glucose-induced tube formation.

### Effect of kaempferol on mRNA expression level of VEGF and PGF and on secreted VEGF and PGF induced by high glucose

**Figure 3:**
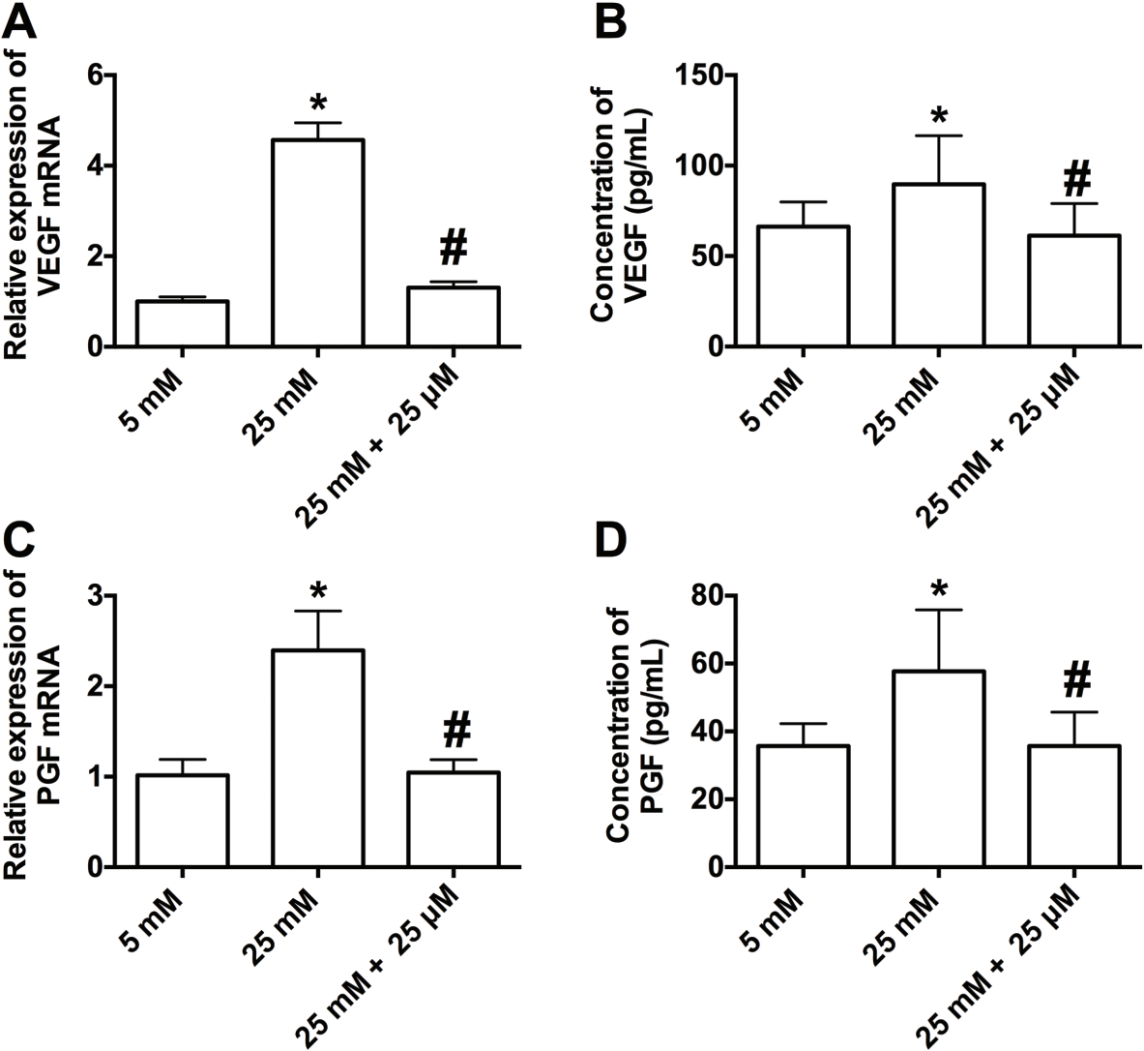
Effect of kaempferol on the mRNA expression level of (*A*) vascular endothelial growth factor (VEGF) and (*C*) placenta growth factor (PGF) in human retinal endothelial cells (HRECs) treated with high glucose (25 mM) alone or high glucose plus kaempferol (25 µM) for 24 h by qRT-PCR. Protein concentrations of VEGF (*B*) and PGF (*D*) in the media were measured by ELISA. Data are reported as means±SD (n=3). *P<0.05 compared to 5 mM; ^#^P<0.05 compared to 25 mM (one-way ANOVA, followed by Dunnett's multiple comparison test).

qRT-PCR results showed that co-treatment with 25 mM high glucose and 25 µM kaempferol suppressed the mRNA expression levels of VEGF and PGF compared to treatment with 25 mM high glucose alone (Figure 3A and B). ELISA assay results showed that the protein concentrations of secreted VEGF and PGF in the media were significantly suppressed by co-treatment with 25 mM high glucose and 25 µM kaempferol compared to treatment with 25 mM high glucose alone (Figure 3C and D).

### Effect of kaempferol on high glucose-induced PI3K expression and Erk1/2, Src, and Akt1 activation

**Figure 4:**
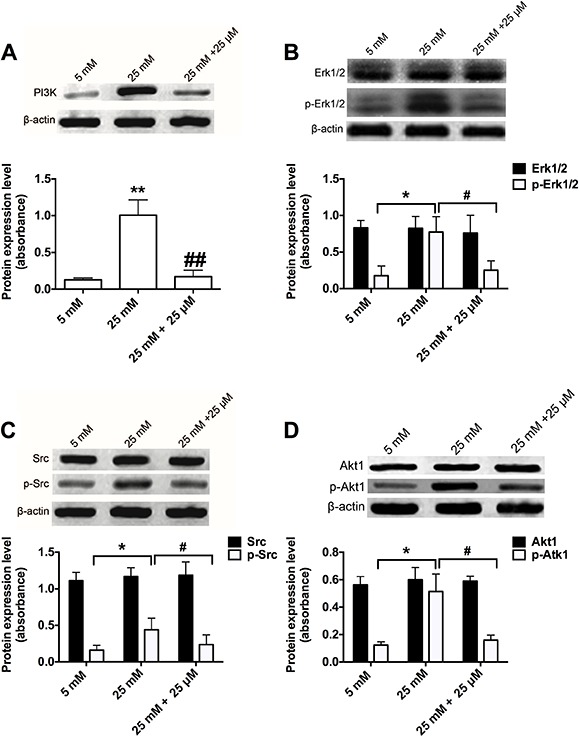
Effects of kaempferol on high glucose-induced PI3K expression and Erk1/2, Src, and AKT1 activation. After human retinal endothelial cells (HRECs) were treated with high glucose (25 mM) alone or high glucose plus kaempferol (25 µM) for 24 h, the protein levels of PI3K (*A*), Erk1/2, p-Erk1/2 (*B*), Src, p-Scr (*C*), AKT1, and p-AKT1 (*D*) were determined by western blot. Data are reported as means±SD (n=3). *P<0.05, **P<0.01 compared to 5 mM. ^#^P<0.05, ^# #^P<0.01 compared to 25 mM (one-way ANOVA, followed by Dunnett's multiple comparison test).

To determine whether kaempferol affects VEGF signaling pathways induced by high glucose, the protein expression of PI3K, and the activation of the downstream factors ERK1/2, Src, and Akt1 were determined by western blot. When HRECs were under 25 mM high glucose condition, the protein expression of PI3K was increased, and ERK1/2, Src, and Akt1 were activated via protein phosphorylation; the levels of total protein were not affected. Treatment with 25 µM kaempferol for 24 h inhibited the protein expression of PI3K and the activation of Erk1/2, Src, and Akt1 induced by high glucose (Figure 4).

## Discussion

In the present study, we demonstrated for the first time that kaempferol suppressed cell proliferation, migration and tube formation of HRECs under high glucose condition, and the anti-angiogenic effect of kaempferol may be mediated via suppressing mRNA and protein expression levels of VEGF and PGF. Further studies demonstrated that kaempferol suppressed high glucose-induced PI3K expression and Erk1/2, Src, and Akt1 activation.

VEGF has been considered to be an important factor in the pathogenesis of DR in response to high glucose or other stimulus, and it can activate VEGFR-1 and -2, which triggers a signaling cascade promoting endothelial cells migration and sprouting (1,7). PGF that upregulates the expression of VEGFA and other angiogenic factors has been shown to be associated with ischemia or wound stimulated angiogenesis and tumor angiogenesis (23). Therefore, VEGF and PGF may be useful targets for the treatment of DR. In the present study, we observed that high glucose stimulation of HRECs increased the mRNA expression of VEGF and PGF as well as the concentrations of secreted VEGF and PGF protein, which is consistent with previous findings (10,11). Similar to previous reports (6), the *in vitro* functional studies further showed that high glucose induced migration and tube formation of HRECs. After establishment of the *in vitro* cell model, we further investigated the anti-angiogenic effects of kaempferol and its underlying molecular mechanisms.

A recent study suggested an anti-angiogenic role of kaempferol in cancer progression. For instance, kaempferol inhibited angiogenesis and VEGF expression through both hypoxia-inducible factor dependent and independent pathways in human ovarian cancer cells (24). In the present study, kaempferol had an anti-angiogenic effect on HRECs, which suppressed migration and tube formation induced by high glucose. The anti-angiogenic effect of kaempferol in HRECs may be mediated via down-regulation of VEGF and PGF, as kaempferol treatment suppressed the expression levels of both VEGF and PGF under high glucose condition.

Pharmacologically, kaempferol has been shown to be an estrogen-related receptor α (ERRα) inverse agonist, which inhibits the activity of ERRα (25), which could cause a decrease in VEGF expression (26). Collectively, it is likely that kaempferol suppressed VEGF expression via targeting ERRα signaling in HRECs, and further studies should be performed to confirm this hypothesis. In future studies, more doses of kaempferol can be tested to further confirm its beneficial effects on DR. Also of scientific interest is the comparison between the beneficial effects of kaempferol and other natural products on DR.

Angiogenesis is a complex and multistep process. Endothelial cell migration is the initial step in angiogenesis, followed by the endothelial cells differentiation into a capillary-like network (27). In the angiogenesis process, interactions of VEGF and PGF with its receptors trigger intracellular signal pathways via activating Src, PI3K/Akt1and Erks (28,29), which in turn affects the angiogenesis of endothelial cells. For the first time, our results demonstrated that kaempferol treatment inhibited high glucose-induced expression of PI3K and the phosphorylation of Src, Akt1 and Erk1/2, which suggested that the anti-angiogenic effect of kaempferol on HRECs under high glucose condition is mediated by downregulation of PI3K and inactivation of Src, Akt1 and Erk1/2 signaling.

In conclusion, our study indicates that kaempferol suppressed angiogenesis of HRECs by targeting VEGF and PGF to inhibit the activation of Src-Akt1-Erk1/2. These results suggest that kaempferol may be a potential drug for better management of DR. Further *in vivo* studies are necessary to confirm the anti-angiogenic effect of kaempferol and to explore its underlying mechanisms.
